# Significant benefits in survival by the use of surgery combined with radiotherapy for retroperitoneal soft tissue sarcoma

**DOI:** 10.1186/s13014-017-0769-0

**Published:** 2017-01-26

**Authors:** Sven Hager, Frank Makowiec, Karl Henne, Ulrich T. Hopt, Uwe A. Wittel

**Affiliations:** 1 Department of General- and Visceral Surgery, Universitätsklinik Freiburg, Hugstetter Str. 55, 79106 Freiburg, Germany; 2 Department of Radiation Oncology, Universitätsklinik Freiburg, Hugstetter Str. 55, Freiburg, 79106 Germany

**Keywords:** Retroperitoneal soft tissue sarcoma, Surgery, Radiotherapy, Radiation therapy, Recurrence

## Abstract

**Background:**

To report the effect of intraoperative electron beam radiotherapy (IOERT) and external beam radiotherapy (EBRT) in addition to surgery as well as to evaluate the role of resectable local recurrence for long-term prognosis.

**Methods:**

In 53 patients who underwent surgery for retroperitoneal soft tissue sarcoma (RSTS) from 2001 to 2014 prognostic and epidemiologic factors were reviewed retrospectively to analyze their impact on survival and recurrence.

**Results:**

Twenty three patients (50%) had surgery plus radiotherapy, 23 (50%) had surgery only. Histology showed 73.9% *liposarcoma*, 15.2% *leiomyosarcoma* and 6.5% *pleomorphic undifferentiated sarcoma* respectively. *Low grade sarcoma* were observed in 52.2%, *high grade sarcoma* in 47.8%. The latter showed a trend towards a decreased 5-year survival rate (*p* = 0.125). Margin status was: R0: 60.9%, R1: 23.9%, R2: 15.2%; leading to significant changes in 5-year survival rate (R0: 77.6%; R1: 70.0%; R2: 42.9%; *p* = 0.03). Age younger than 55 years significantly improved 5-year survival rate (*p* = 0.039). Patients receiving resection of multiple sarcoma recurrence showed an almost identical improved 5-year survival rate compared to patients without recurrence (no recurrence: 100.0%; single recurrence: 35.0%; multiple recurrence: 91.7%; *p* = 0.001). Surgery plus radiotherapy led to significantly improved survival (*p* = 0.04).

**Conclusions:**

There is a significant benefit in terms of 5-year survival after surgery plus some form of radiotherapy and a good prognosis for patients when the recurrence from RSTS was resected. Age older than 55 years and incomplete resection lowered 5-year survival rate significantly.

## Background

Soft-tissue sarcoma are rare tumor entities arising from mesenchymal tissue. They account for less than 1% of all solid tumors. About 85% of retroperitoneal tumors are malignant and among those Retroperitoneal Soft Tissue Sarcoma (RSTS) make about one third [1, 2]. Histologically there are 3 predominant subtypes: liposarcoma (26–64.5%), leiomyosarcoma (13.2–31%) and pleomorphic undifferentiated sarcoma (PUS) (7–27%) [2–10].

Due to their anatomic origin the clinical presentation of RSTS is typically inapparent and characterized by late symptoms and a large tumor size at the time of diagnosis. Consecutively vital structures and organs are already involved or at risk which make the complete resection difficult to achieve [3].

In current literature surgical resection is the mainstay of treatment to achieve a long-term disease free survival or cure. The rate of complete resection ranges from 41.8 to 76% [2–10]. Local recurrence rate is high even after complete removal of the tumor. Among the treatment modalities chemotherapy plays a minor role in adult patients, whereas it has its benefits in bone and soft tissue sarcoma (Ewing sarcoma, rhabdomyosarcoma, osteosarcoma) that commonly occur in children [11]. The rationale for applying radiotherapy in addition to surgery comes from small randomized controlled trials showing evidence that radiotherapy enhances local control and resectability in soft tissue sarcoma of the limbs and RSTS without affecting survival [12–14]. In other retrospective studies these positive effects on local control have been demonstrated in RSTS as well. Some studies even propose a better overall survival [7, 8, 10, 15–17]. On the other hand opponents argue that the value of radiotherapy remains questionable given that in most studies no survival difference was detected [4, 18].

In this study prognostic and epidemiologic factors as well as the 5-year-survival-rate of 53 patients after surgery with or without radiotherapy for primary and recurrent RSTS was assessed.

## Materials and methods

Approval of the local Ethical Committee as well as the institutional review board was obtained before commencing this study. In this series data from 53 patients who underwent surgery under curative intention for RSTS from 2001 to 2014 in a single institution *(Department of General- and Visceral Surgery, University of Freiburg)* were reviewed retrospectively. 7 patients were excluded due to incomplete data or different tumor entity in postoperative pathological report. For the remaining 46 patients with primary RSTS demographic characteristics, clinicopathological parameters and treatment variables were obtained in medical records, cancer registry entries, radiology, pathology and operative reports.

The tumor characterization (TNM) was done in accordance with the seventh edition of the American Joint Committee on Cancer (AJCC) staging system for soft tissue sarcoma. Histopathologic subtypes were subdivided into liposarcoma, leiomyosarcoma, pleomorphic undifferentiated sarcoma (PUS) and others. The histologic grading was performed according to the FNLCC (Fédération Nationale des Centres de Lutte Contre le Cancer) grading system whereupon FNLCC grade 1 and 2 were summed up as “*low-grade-sarcoma*” whereas FNLCC grade 3 was regarded as “*high-grade-sarcoma*”. Tumor size was documented by its greatest diameter into the groups *<5 cm, 5–10 cm, 10–20 cm, >20 cm*.

Treatment variables analyzed included margin status, local or visceral resection, chemotherapy and intraoperative (IOERT) or perioperative radiotherapy: Margin status was defined as R0 for microscopically tumor free margins (complete resection), R1 for microscopically infiltrated tumor margins (incomplete resection) and R2 for macroscopically infiltrated tumor margins or residual tumor at the operation site (incomplete resection).

Primary RSTS was defined as a tumor before treatment. For statistical analysis no further differentiation was made between local or distant recurrence. So recurrence was defined as histologically proven recurrence in close proximity or distant from the primary tumor site. Tumor growth after R2 resected tumors was not included. Perioperative morbidity was not assessed in this study. Perioperative mortality was defined as death within 30 days after surgery.

### Surgical technique

Concerning our surgical approach we performed an aggressive form of treatment established by Gronchi and colleagues [8]. They could see lower rates of local and distant recurrence after performing radical liberal en bloc resection of all surrounding tissues and adjacent organs in a proximity of 1 to 2 cm from the tumor surface. Resection includes a locoregional peritonectomy which is often accompanied by mainly removal of adjacent organs which are fully or partially located in the retroperitoneum (kidneys, colon, pancreas, duodenum, psoas muscle, diaphragm) when located close to the tumor. With bigger tumors the extent of resection easily exceeds the sole resection of retroperitoneally located viscera. Local resection was defined as resection of the tumor with adjacent soft tissue but without visceral organs. Visceral or multivisceral resection of the tumor with one or more adjacent organs were summarized to one group.

### Additional therapy

The decision whether to receive radiotherapy/chemotherapy or not was made by a multidisciplinary team of surgeons, oncologists and radiotherapists without randomization in accordance to the surrounding tissue and expected morbidity due to radiation.

### Chemotherapy

Chemotherapy neoadjuvant and/or adjuvant included cisplatin, ifosfamide, doxorubicin, adriamycin, paclitaxel, etoposid or epirubicin (VIC/VIP-E protocol).

### Radiotherapy

Radiotherapy included either neoadjuvant external beam radiotherapy (naEBRT), intraoperative (IOERT) or adjuvant external beam radiotherapy (aEBRT). Sometimes treatment included the combination of two or all three modalities. For statistical analysis all patients receiving some sort of radiotherapy whether neoadjuvant, intraoperative or adjuvant were summarized to one group (S + RT).

An indication for IOERT was given if the tumor was located in close proximity to irresectable structures and if an R0 resection could not be anticipated safely prior to operation. In uncertain cases operations were planned with IOERT in stand-by.

All patients that were considered for IOERT had surgery in a specific operating theatre with an integrated linear accelerator (Siemens Mevatron ME®, Siemens Healthcare GmbH, Erlangen, Germany). Prior to IOERT our surgical team radically removed the tumor an adjacent organs if necessary, marked tumor margins and send the specimen to the pathologic for intraoperative frozen section. In cases where the intraoperative situation evolved with adequate distance to the tumor and if our pathologists confirmed intraoperatively tumor free margins with a thick layer of healthy tissue in between the indication for IOERT was abandoned in consent with our attendant radiation therapists.

When receiving IOERT the resection site was explained to radiation therapist by the surgeon. After receiving the results of the frozen section further resections were made in case of R1 margins to achieve the best possible resection status. The surgeon manually attached in correlation with size and form of the tumor bed a suitable applicator to the OR-table and aligned it to match the IOERT field with the tumor cavity. For protecting uninvolved surrounding tissue lead shields (5 mm) were applied. Attached to a mobile operation table the patient was transferred underneath the linear accelerator and consecutively the applicator was docked by a laser air-docking system (100 cm focus-to-surface distance). The typical IOERT dose was 15 Gy, using a electron energy of 6 MeV (depth of 1.6 to 1.9 cm, 90% isodose level). Higher radiation doses up to 20 Gy were applied due to suspected R1 resection.

Before applying external beam radiation therapy (EBRT) tumor volumes and organs at risk were defined on a case to case basis from a planning CT scan or MRI due to the individual characteristics of RSTS in localization and tumor infiltration. EBRT was applied in classic 4 field box technique (Elekta Synergy®, Elekta Instrument AB Stockholm, Stockholm, Sweden) or in form of volumetric modulated arc therapy (VMAT) (Rapid Arc®, Varian Medical Systems, Palo Alto, CA, USA).

Treatment planning for volumetric-modulated arc therapy was done with the EclipseTM treatment planning system (Varian Medical Systems, Palo Alto, CA, USA). A 1–1.5 cm margin was added to the planning target volume. Beam energy was 15 MeV.

The treatment planning system Oncentra MasterPlan® (OTP, Nucletron BV (Elekta), Veenendaal, The Netherlands) was used for 4 field box radiotherapy. A 1 cm margin was added to the planning target volume, which was generated from preinterventional MRI or CT-Scans. Single beam energy was 6, 15 or18 MeV. Beams-eye projections of the planning target volume were generated through multi-leaf collimators. The field controls were done with cone beam CTs.

### Statistical analysis

Statistical analysis was performed with SPSS® (Statistical Package for Social Sciences, 22.0, Chicago, IL, USA). Survival and Progression Free Survival (PFS) were calculated using the Kaplan–Meier method and univariate analysis was performed with log-rank test and the Gehan-Breslow-Wilcoxon method. In addition univariate analysis was performed using chi-square test und Fisher’s exact test. Deaths were confirmed by our tumor registry. There was no further analysis of the causes for death. Patients with R2 margins were excluded from PFS analysis as well as the analysis of recurrence due to their persistent disease by definition. *P* values <0.05 were considered to be significant.

To determine independent prognostic factors Cox proportional hazards regression model was used with all factors that were significant (or close to significance *P* < 0,1) in the univariate analysis.

## Results

### Demographic and tumor characteristics

Patient and tumor characteristics of the 46 patients with RSTS included in this study are summarized in Table 1. The median follow-up was 55.51 months (range 7–148 months).

**Table 1:** Demographic, Tumor and Treatment Characteristics of 46 patients with RSTS who underwent surgical resection at the *Department of General- and Visceral Surgery, University of Freiburg* from 2001 to 2014

	N (% of total)
Age (years)	
< 55	20 (43.5%)
> 55	26 (56.5%)
Gender	
Male	25 (54.3%)
Female	21 (45.7%)
Presentation	
Primary	24 (52.2%)
Recurrent	22 (47.8%)
Tumor Grade	
High	22 (47.8%)
Low	24 (52.2%)
Histologic subtype	
Liposarcoma	34 (73.9%)
Leiomyosarcoma	7 (15.2%)
PUS	3 (6.5%)
Other	2 (4.3%)
Tumor size	
< 5 cm	2 (4.3%)
5–10 cm	7 (15.2%)
10–20 cm	13 (28.3%)
> 20 cm	24 (52.2%)
Resection status	
R0	28 (60.9%)
R1	11 (23.9%)
R2	7 (15.2%)
Local resection	18 (29.1%)
Visceral resection	28 (60.9%)
Radiotherapy	
Radiotherapy + surgery	23 (50%)
No radiotherapy	23 (50%)
Chemotherapy	
Neoadjuvant	2 (10.9%)
Adjuvant	5 (4.3%)
Both	1 (2.2%)
None	39 (84.8%)

*N* number of patients

Among those were 25 men (54.3%) and 21 women (45.7%). The median age was 59.9 years (range: 33–83 years). 20 of the patients were younger than 55 years (43.5%) while 26 of the patients exceeded 55 years of age (56.5%).

Histology in 73.9% of the patients showed *liposarcoma*, while 15.2% and 6.5% of the patients showed *leiomyosarcoma* and *pleomorphic undifferentiated sarcoma (PUS)* respectively. In 2 cases definitive histologic classification to one of those subtypes was not possible due to poor tumor differentiation and was classified as malignant pleomorphic mesenchymal tumors. *Low-grade-sarcoma* was observed in 52.2% of patients while *high-grade-sarcoma* was observed in 47.8% of patients.

In 4.3% of patients (*n* = 2) tumor size was <5 cm, in 15.2% (*n* = 7) tumor size was 5–10 cm, in 28.3% (*n* = 13) tumor size was 10–20 cm and in 52.2% (*n* = 24) tumor size exceeded 20 cm at primary diagnosis.

Median overall-survival for the 46 patients was not yet reached. Of all patients 71.2% were alive after 5 years. The mean overall-survival was 112.1 months. There was no significant influence on 5-year-survival and *PFS* by gender, histological subtype or size of primary tumor.

### Age

We could see significant benefits in survival of patients younger than 55 years (89.59% vs. 63.1%; *p* = 0.039) (Table 2). Influence of age on *PFS* could not be shown (*p* = 0.740). Multivariate analysis showed a trend that age could be an independent prognostic factor without reaching significance (*p* = 0.121).

**Table 2:** Univariate analysis of prognostic factors influencing 5-year-survival-rate/Progression free survival in 46 patients with retroperitoneal soft tissue sarcoma

	All patients *n* = 46					After complete resection *n* = 39				
	Suvival rate					Sarcoma recurrence				
	*N*	Events (%)	5-YSR (%)	SD	*P*	*N*	Events (%)	PFS (%)	SD	*P*
Age (years)					0.039					0.740
< 55	20	2 (10.0%)	89.5%	7.0		19	10 (52.6%)	37.3%	13.3	
> 55	26	9 (34.6%)	63.1%	10.5		20	9 (45.0%)	27.7%	20.5	
Gender					0.643					0.112
Male	25	5 (20.0%)	75%	10.2		21	8 (38.1%)	40.9%	16.3	
Female	21	6 (28.6%)	66.8%	11.2		18	11 (61.1%)	26.6%	13.6	
Presentation					0.001					0.753
No recurrence	17	0 (0%)	100%	0						
Single recurrence	10	6 (60.0%)	40%	9.5						
Multiple recurrence	12	1 (8.3%)	91.7%	8.0						
Tumor Grade					0.125					
High	22	8 (36.4%)	59.4%	11.3		17	9 (52.9%)	38.2%	14.5	
Low	24	3 (12.5%)	85.6%	7.7		22	10 (45.5%)	29.0%	15.6	
Tumor size					0.711					0.711
< 20 cm	22	5 (22.7%)	72.1%	10.9		19	10 (52.6%)	37.2%	14.0	
> 20 cm	24	6 (25.0%)	70.9%	10.2		20	9 (45.0%)	30.1%	16.2	
Resection status					0.03					0.212
R0	28	4 (14.3%)	77.6%	10.6		28	11 (39.3%)	45.4%	15.1	
R1	11	3 (27.3%)	70.0%	14.5		11	8 (72.7%)	21.2%	13.2	
R2	7	4 (57.1%)	42.9%	18.7						
Local resection	18	3 (16.7%)	80.8%	9.9	0.267	13	6 (46.2%)	44.1%	16.6	0.582
Visceral resection	28	8 (28.6%)	65.2%	10.1		26	13 (50.0%)	26.5%	14.3	
Radiotherapy					0.060					0.362
S + RT	23	3 (13.0%)	82.3%	9.8		22	9 (40.9%)	41.2%	18.3	
SO	23	8 (34.8%)	58.6%	11.4		17	10 (58.8%)	26.8%	12.6	
Chemotherapy					0.344					0.899
Yes	7	3 (42.6%)	57.1%	18.7		5	3 (60.0%)	40.0%	21.9	
None	39	8 (20.5%)	74.1%	8.2		34	16 (47.1%)	28.7%	12.6	

*N* number of patients*, Events* number of deaths and/or recurrences*, 5-YSR* 5 year survival rate*, PFS* progression free survival*, SD* standard deviation*, P p-*value*, S + RT* surgery plus radiotherapy*, SO* surgery only

### Grading

*High-grade-sarcoma* showed a trend in decreased 5-year-survival with 59.4% compared to *low-grade-tumors* with 85.6% (*p* = 0.125) (Table 2). There was no difference in *PFS* (*p* = 0.753).

### Surgery

Liberal en bloc resection of the adjacent tissues or organs was performed after abdominal and retroperitoneal exploration. Altogether 99 surgical resections were performed in 46 patients of whom 22 patients in part with multiple tumor recurrence had repetitive surgery ranging from 2 to 7 operations. After primary surgery 28 patients (60.9%) had a R0-resection, 11 patients (23.9%) had a R1-resection and 7 patients (15.2%) had a R2-resection. Consecutively patients with incomplete resection had a higher rate of local or distant recurrence (81.8%, *n* = 9/11) compared to patients with complete resection (46.4%, *n* = 13/28). 18.2% of patients with incomplete resection had no recurrence detected most likely due to early death within 1 year after surgery, or due to short follow up time of approximately 1 year. Overall there was tumor recurrence in 22 patients (47.8%). Among those 22 patients 10 had a single recurrence (SR, 21.7%) and 12 patients had multiple recurrences (MR, 26.1%). In all 22 patients a second surgical procedure was performed achieving a secondary complete resection in 59.1%. In 31.8% (*n* = 7) of the cases a R1-status and in 9.1% (*n* = 2) of the cases a R2-status was attained.

In 39.1% of patients a local resection was possible, in 60.9% one or more contiguous organs or structures had to be removed to achieve a R0-status (kidney *n =* 19, psoas muscle *n* = 17, colon/rectum *n =* 16, small intestine *n = 11*, parts of the abdominal wall *n = 10,* diaphragm *n = 9,* spleen *n =* 6, gallbladder *n =* 6, adrenal gland *n = 5,* liver *n =* 5, partial resection of V. cava *n = 5,* pancreas *n =* 4, uterus *n = 4*, bladder *n =* 4, stomach *n = 2*). Morbidity was not addressed in this study. Among the 46 included patients there was no perioperative mortality. One patient excluded from the study group due to missing data about primary tumor characteristics died perioperatively. So mortality of the whole collective of 53 patients was 1.9%.

### Margin status

**Fig. 1:**
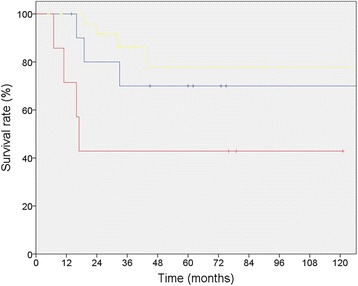
Sarcoma specific 5 year-survival of 46 patients with RSTS who underwent surgical resection divided by R-status (R0-margin [*yellow*] 77.6% vs. R1-margin [*blue*] 70.0% vs. R2-margin [*red*] 42.9%; *p* = 0.03)

R-Status lead to significant changes in 5-year-survival, with a R2-resection resulting in decreased survival (*p* = 0,03) (Table 2, Fig. 1). Multivariate analysis showed that R-Status is an independent prognostic factor for survival (*p* = 0.027) while *PFS* was statistically not significantly influenced (*p* = 0,212).

**Fig. 2:**
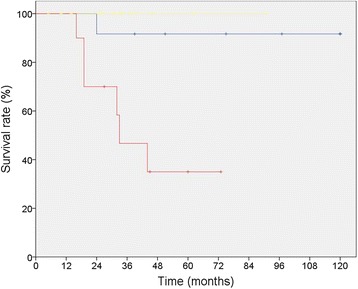
Sarcoma specific 5 year-survival of 46 patients with RSTS who underwent surgical resection divided by type of recurrence (no recurrence [*yellow*] 100.0% vs. single recurrence [*red*] 40.0% vs. multiple recurrences [*blue*] 91.7%, *p* = 0.001)

Five-year-survival-rate and *PFS* was not influenced by local resection compared to resections including involved adjacent organs (Table 2). As long as recurrence could be resected there was no influence on survival provided that tumor recurrence was resected. This resulted in an almost identical 5-year-survival of patients with multiple recurrence compared to patients without recurrence (91.7% vs. 100.0%; *p* = 0.317), whereas 5-year-survival-rate of patients with a single recurrence was significantly worse in comparison (35.0%0; *p* = 0.001) (Table 2, Fig. 2). In patients where repeated recurrences could be resected tumor biology was characterized by slow tumor growth which led to a 5-year-overall-survival of all patients. This was independent of the grading of the sarcoma, since of 11 patients with multiple recurrences, 5 patients had *high-grade-sarcoma* (46.5%) and 6 patients had *low-grade-sarcoma* (54.5%). Only one of these patients died within 5 years – having a *high-grade-sarcoma*.

### Radiotherapy

**Fig. 3:**
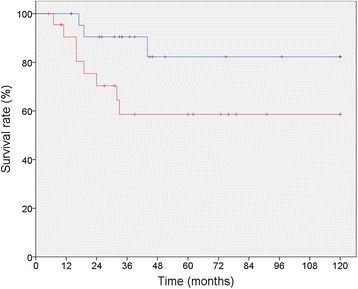
Sarcoma specific 5-year-survival of 46 patients with RSTS who underwent surgical resection with or without radiotherapy (SO = surgery only [*red*] 58.6% vs. S + RT = surgery plus radiotherapy [*blue*] 82.3%, *p* = 0.043)

Twenty three (50%) patients received additional naEBRT, IOERT or aEBRT in combination with their primary or consecutive surgery. Patients receiving some form of radiotherapy in combination with surgery (S + RT) showed improved survival compared to patients receiving surgery only (SO) (82.3% vs. 58.6% *p* = 0,04) (Table 2, Fig. 3). A subset analysis for comparison of the characteristics of patients undergoing *SO* vs. *S + RT* showed that there is no significant selection bias between these two groups. By multivariate analysis overall-survival was not affected by receiving radiotherapy (*p* = 0.251).

In the group of 21 patients with recurrences 11 patients received radiotherapy. Among those patients all had local recurrences only 2 patients developed distant pulmonary metastasis in the later course of their disease. The mean time until recurrence of the disease was 40.9 months in the surgery plus radiotherapy group vs 32.8 months in the surgery only group. So PFS was slightly extended in the surgery plus radiotherapy group without being significant (*p* = 0.362) which indirectly suggests that there probably are indirect benefits in local control. We did not perform exact analysis of local control because the timing and application of radiotherapy was very heterogeneous and the individual effect of each form of radiotherapy could not be differentiated further.

All 11 patients with tumor recurrences in the S + RT group had local recurrences. In patients receiving some form of EBRT +/− IOERT all local recurrences were within the former tumor site or close to its margins meaning that they were in field. Only 2 patients developed distant pulmonary metastasis in addition to their local recurrence in the later course of their disease. For patients who have received IOERT we cannot reproduce the exact radiation fields due to the technique (as described above) because the surgeon individually adapts a suitable applicator to the intraoperative tumor bed without prior image based planning of the exact field of radiation. After IOERT and surgery the resection cavity changes in shape and size after surgically closing the wound. So the postoperative correlation if a loco regional recurrence is exactly in field or probably not is difficult to do.

### Chemotherapy

Eight patients (20%) received neoadjuvant and/or adjuvant chemotherapy, including cisplatin, ifosfamide, doxorubicin, adriamycin, paclitaxel, etoposid and epirubicin (VIC/VIP-E protocol). Receiving some form of chemotherapy in combination with surgery had no influence on overall survival (*p* = 0.344) and on *PFS* (*p* = 0.899) (Table 2).

## Discussion

A major issue of studies concerning the treatment of retroperitoneal sarcoma is that randomized controlled studies are rare due to small patient numbers in general. Accordingly most data – as well as in this study - was collected in retrospective surveys. Therefore the limitations of retrospective study design do apply for most findings.

Unlike most solid organ derived tumors of visceral cavity the individual prognosis of RSTS is directly linked to the site of origin of the primary tumor and its surroundings. Id est the extent of an indicated surgical resection is not standardized among individual cases and f.e. a tumor of identical histology and size is treated differently due to its origin and adjacent vital structures.

Related to this consideration established prognostic factors for overall survival in patients with retroperitoneal sarcoma comprise margin status, grading, histological subtype and tumor size [4, 5, 14, 19, 20].

In larger studies high grade tumors are related to a poorer 5-year-survival-rate [2, 3, 5, 9, 19]. We could not reproduce this effect significantly. This is maybe due to small patient numbers in this study. In all 46 patients *high-grade-sarcoma* showed a trend in decreased 5-year-survival with 59.4% compared to *low-grade-tumors* with 85.6% (*p* = 0.125). There was no difference in *PFS* (*p* = 0.753). Either gender or tumor size did prove to be significant prognostic factors for survival. Interestingly we found age younger than 55 years to be significantly beneficial for 5-year-survival in the univariate analysis. Reasons for this observation remain notional. There was no further analysis of the causes for death. So no conclusions whether death was sarcoma related or not could be made. A lack of comorbidities in the group “age <55 years” could be a possible explanation. In a subset analysis there was no selection bias between these two groups.

### Surgery

The complete surgical resection is the main treatment modality in patients with retroperitoneal sarcoma [3, 9]. With large tumor size at the time of diagnosis and its margins in close proximity to vital structure, complete surgical resection is a challenging goal to achieve. The rate of negative resection margins ranges from 41.8 to 76%, which then result in improved survival [2–10]. In our analysis margin status proved to be an independent prognostic factor for 5-year-survival-rate within the univariate as well as multivariate analysis (*p* = 0,03/*p* = 0,027).

In our cohort we could achieve a complete resection in 60.9% of our patients. Although complete resection can be achieved in most cases, the rate of recurrence is high and varies between 28 and 77% [5, 20]. In our study we could see a *5-year*-*PFS* of only 34.4% ± 10.8%. Despite these discouraging results in *PFS* we achieved a secondary margin free resection in 59.1% (*n* = 13) of the 22 patients with tumor recurrence, similar to Gholami et al. and Van Dalen et al. who demonstrated that a secondary complete resection in patients with sarcoma recurrence could be achieved in most cases resulting in resection rates comparable to those of primary surgery. With subsequent surgery a disease-free-status was achieved and survival was improved [19, 21].

In *low-grade-sarcoma* we could see most patients with a long term survival exceeding the 5-year-range by far even if a complete resection has not been achieved. Interestingly we found in our data that survival of patients with multiple recurrences and patients without recurrence was almost identical resulting in long term survival for individual patients with multiple recurrences throughout the whole observation period. There was no significant selection bias concerning tumor grading within this subgroup.

Congruently Van Dalen et al. could see that in contrast to patients with *high-grade-sarcoma* a large proportion of patients with low-grade-tumors remained alive for a long period. Loco regional and slower growth of low-grade-recurrences as well as better opportunities for repetitive surgical treatment were possible explanations for these findings [22]. Furthermore Petersen et. al. could show that neither primary vs. recurrent status nor tumor grade had a significant impact on survival [23].

A possible explanation for the decreased 5-year-survival in our patients with a single resected recurrence is the higher percentage of cases with leiomyosarcoma, PUS and one malignant pleomorphic mesenchymal tumors within this subgroup (no recurrence: 23.5% (*n* = 4/17); multiple recurrence: 25.0% (*n* = 3/12), single recurrence: 40.0% (*n* = 4/10)) which are associated with an reduced 5 year-survival-rate [9]. In addition the two cases where a secondary complete resection could not be achieved pertain to this subgroup.

Frankly, there is evidence that the prognosis for patients with tumor recurrence that is resectable is not necessarily worse than in patients without recurrence.

Regarding this we think that subsequent surgery even with multiple recurrences over the course of time provides a solid basis for long term survival especially in low-grade-RSTS. Consistent with findings of Colleagues at the Stanford University and the University of Heidelberg we could see that treatment of local recurrences, even on multiple occasions, is effectively possible by subsequent surgical resection [19, 24].

### Radiotherapy

In literature the benefits of radiotherapy in addition to surgery are controversially discussed. Although there is a consensus among most studies that surgery in addition to EBRT with or without IOERT lead to better outcomes regarding local control in cases with primary and recurrent disease or in patients with microscopically incomplete resection as well as reduced risk of sarcoma related death [5, 8, 10, 23, 25–27], there was few data of improved influence on survival [15, 17, 23, 26]. Therefore some colleagues argued that the value of radiotherapy remains questionable particularly if it comes at the expense of toxic complications [4, 18]. Strong arguments for the impact of EBRT on survival are given by a recent study of Nussbaum and colleagues: It is the largest study to date including over 9000 patients analyzed retrospectively for the effect of neoadjuvant or adjuvant radiotherapy on overall survival in patients with RSTS. Both neoadjuvant and adjuvant radiotherapy were associated with improved 5-year-survival compared with surgery only (neoadjuvant: 62% versus 54%, *p* < 0 · 0001; adjuvant: 60% versus 52%, *p* < 0 · 0001) [28].

Severe toxic complications related to radiation after treating RSTS with EBRT and/or IOERT range from 10 to 37%. They include neuropathy, grade 3 and 4 enteritis, hydronephrosis, gastrointestinal fistula, gastric outlet obstruction, bowel obstruction, vaginal fistula, wound complications, abscesses and bleeding [25].

In our study we could see significant benefits on 5-year-survival after S + RT. As mentioned above there was no randomization, which could have had an influence on survival as well as on the risk of recurrence. However patients receiving S + RT showed improved survival without affecting *PFS* in the univariate analysis (S + RT 82.3%, SO 58.6%; *p* = 0,04). But it was not an independent prognostic factor in multivariate analysis (*p* = 0.251). Our findings are congruent with a recent Swedish study as well as with the study of Pierie et al.. They did not only show an improved rate of local control but also significant advantages in 5-year-overall-survival [15, 26].

## Conclusions

Complete surgical resection is an important prognostic variable that can be performed in patients with primary and recurrent sarcoma. Our results show that there is an improved survival even with multiple resections as well as in combination with radiotherapy. To provide evidence in treatment recommendations, larger multicentric or multinational, randomized, controlled trials are imperatively required.

## Abbreviations

5-YSR: 5 year survival rate
aEBRT: Adjuvant external beam radiotherapy
AJCC: American Joint Committee on Cancer
EBRT: External beam radiotherapy
FNLCC: Fédération Nationale des Centres de Lutte Contre le Cancer
IOERT: Intraoperative electron beam radiotherapy
MR: Multiple recurrence
naEBRT: Neoadjuvant external beam radiotherapy
PFS: Progression free survival
PUS: Pleomorphic undifferentiated sarcoma
RSTS: Retroperitoneal soft tissue sarcoma
S + RT: Surgery plus radiotherapy
SD: Standard deviation
SO: Surgery only
SR: Single recurrence
VMAT: Volumetric modulated arc therapy

## References

[1] Jaques DP, Coit DG, Hajdu SI, Brennan MF (1990). Management of primary and recurrent soft-tissue sarcoma of the retroperitoneum. Ann Surg.

[2] Gronchi A, Casali PG, Fiore M, Mariani L, Lo Vullo S, Bertulli R (2004). Retroperitoneal soft tissue sarcomas: patterns of recurrence in 167 patients treated at a single institution. Cancer.

[3] Lewis JJ, Leung D, Woodruff JM, Brennan MF (1998). Retroperitoneal soft-tissue sarcoma: analysis of 500 patients treated and followed at a single institution. Ann Surg.

[4] Van Dalen T, Hoekstra HJ, Van Geel AN, Van Coevorden F, Albus-Lutter C, Slootweg PJ (2001). Locoregional recurrence of retroperitoneal soft tissue sarcoma: second chance of cure for selected patients. Eur J Surg Oncol.

[5] Stoeckle E, Coindre JM, Bonvalot S, Kantor G, Terrier P, Bonichon F (2001). Prognostic factors in retroperitoneal sarcoma: a multivariate analysis of a series of 165 patients of the French Cancer Center Federation Sarcoma Group. Cancer.

[6] Mendenhall WM, Zlotecki RA, Hochwald SN, Hemming AW, Grobmyer SR, Cance WG (2005). Retroperitoneal soft tissue sarcoma. Cancer.

[7] Toulmonde M, Bonvalot S, Meeus P, Stoeckle E, Riou O, Isambert N (2014). Retroperitoneal sarcomas: patterns of care at diagnosis, prognostic factors and focus on main histological subtypes: a multicenter analysis of the French Sarcoma Group. Ann Oncol.

[8] Gronchi A, Lo Vullo S, Fiore M, Mussi C, Stacchiotti S, Collini P (2009). Aggressive surgical policies in a retrospectively reviewed single-institution case series of retroperitoneal soft tissue sarcoma patients. J Clin Oncol Off J Am Soc Clin Oncol.

[9] Bonvalot S, Rivoire M, Castaing M, Stoeckle E, Le Cesne A, Blay JY, Laplanche A (2009). Primary retroperitoneal sarcomas: a multivariate analysis of surgical factors associated with local control. J Clin Oncol Off J Am Soc Clin Oncol.

[10] Sampath S, Hitchcock YJ, Shrieve DC, Randall RL, Schultheiss TE, Wong JY (2010). Radiotherapy and extent of surgical resection in retroperitoneal soft-tissue sarcoma: multi-institutional analysis of 261 patients. J Surg Oncol.

[11] Pisters PW, O’Sullivan B, Maki RG (2007). Evidence-based recommendations for local therapy for soft tissue sarcomas. J Clin Oncol Off J Am Soc Clin Oncol.

[12] Pisters PW, Harrison LB, Leung DH, Woodruff JM, Casper ES, Brennan MF (1996). Long-term results of a prospective randomized trial of adjuvant brachytherapy in soft tissue sarcoma. J Clin Oncol Off J Am Soc Clin Oncol.

[13] Sindelar WF, Kinsella TJ, Chen PW, DeLaney TF, Tepper JE, Rosenberg SA, Glatstein E (1993). Intraoperative radiotherapy in retroperitoneal sarcomas. Final results of a prospective, randomized, clinical trial. Arch Surg.

[14] Yang JC, Chang AE, Baker AR, Sindelar WF, Danforth DN, Topalian SL (1998). Randomized prospective study of the benefit of adjuvant radiation therapy in the treatment of soft tissue sarcomas of the extremity. J Clin Oncol Off J Am Soc Clin Oncol.

[15] Trovik LH, Ovrebo K, Almquist M, Haugland HK, Rissler P, Eide J (2014). Adjuvant radiotherapy in retroperitoneal sarcomas. A Scandinavian Sarcoma Group study of 97 patients. Acta Oncol.

[16] Zhou Z, McDade TP, Simons JP, Ng SC, Lambert LA, Whalen GF (2010). Surgery and radiotherapy for retroperitoneal and abdominal sarcoma: both necessary and sufficient. Arch Surg.

[17] Stucky CC, Wasif N, Ashman JB, Pockaj BA, Gunderson LL, Gray RJ (2014). Excellent local control with preoperative radiation therapy, surgical resection, and intra-operative electron radiation therapy for retroperitoneal sarcoma. J Surg Oncol.

[18] Heslin MJ, Lewis JJ, Nadler E, Newman E, Woodruff JM, Casper ES (1997). Prognostic factors associated with long-term survival for retroperitoneal sarcoma: implications for management. J Clin Oncol Off J Am Soc Clin Oncol.

[19] Gholami S, Jacobs CD, Kapp DS, Parast LM, Norton JA (2009). The value of surgery for retroperitoneal sarcoma. Sarcoma.

[20] Bonvalot S, Miceli R, Berselli M, Causeret S, Colombo C, Mariani L (2010). Aggressive surgery in retroperitoneal soft tissue sarcoma carried out at high-volume centers is safe and is associated with improved local control. Ann Surg Oncol.

[21] Van Dalen T, Hennipman A, Van Coevorden F, Hoekstra HJ, Van Geel BN, Slootweg P (2004). Evaluation of a clinically applicable post-surgical classification system for primary retroperitoneal soft-tissue sarcoma. Ann Surg Oncol.

[22] Van Dalen T, Plooij JM, Van Coevorden F, Van Geel AN, Hoekstra HJ, Albus-Lutter C (2007). Long-term prognosis of primary retroperitoneal soft tissue sarcoma. Eur J Surg Oncol.

[23] Petersen IA, Haddock MG, Donohue JH, Nagorney DM, Grill JP, Sargent DJ, Gunderson LL (2002). Use of intraoperative electron beam radiotherapy in the management of retroperitoneal soft tissue sarcomas. Int J Radiat Oncol Biol Phys.

[24] Lehnert T, Cardona S, Hinz U, Willeke F, Mechtersheimer G, Treiber M (2009). Primary and locally recurrent retroperitoneal soft-tissue sarcoma: local control and survival. Eur J Surg Oncol.

[25] van de Voorde L, Delrue L, Van Eijkeren M, De Meerleer G (2011). Radiotherapy and surgery-an indispensable duo in the treatment of retroperitoneal sarcoma. Cancer.

[26] Pierie JP, Betensky RA, Choudry U, Willett CG, Souba WW, Ott MJ (2006). Outcomes in a series of 103 retroperitoneal sarcomas. Eur J Surg Oncol.

[27] Yoon SS, Chen YL, Kirsch DG, Maduekwe UN, Rosenberg AE, Nielsen GP (2010). Proton-beam, intensity-modulated, and/or intraoperative electron radiation therapy combined with aggressive anterior surgical resection for retroperitoneal sarcomas. Ann Surg Oncol.

[28] Nussbaum DP, Rushing CN, Lane WO, Cardona DM, Kirsch DG, Peterson BL, Blazer DG (2016). Preoperative or postoperative radiotherapy versus surgery alone for retroperitoneal sarcoma: a case–control, propensity score-matched analysis of a nationwide clinical oncology database. Lancet Oncol.

